# 1182. Risk factors for colonization with extended-spectrum cephalosporin resistant and carbapenem resistant Enterobacterales among community adults, Bangladesh: An Antibiotic Resistance in Communities and Hospitals (ARCH) study

**DOI:** 10.1093/ofid/ofac492.1017

**Published:** 2022-12-15

**Authors:** Syeda Mah-E-Muneer, Fahmida Chowdhury, Kamal Hossain, Ulzii-Orshikh Luvsansharav, Md Zakiul Hassan, Rachel Mann Smith, Ashley R Styczynski

**Affiliations:** icddr,b, Dhaka, Dhaka, Bangladesh; icddr,b, Dhaka, Dhaka, Bangladesh; icddr,b, Dhaka, Dhaka, Bangladesh; CDC, Atlanta, Georgia; icddr,b, Dhaka, Dhaka, Bangladesh; CDC, Atlanta, Georgia; Centers for Disease Control and Prevention, Atlanta, Georgia

## Abstract

**Background:**

Antimicrobial resistant (AMR) organisms are an increasing global health threat that are spreading within communities. Understanding the risk factors for colonization with AMR organisms is critical for implementing prevention and control strategies, particularly in resource-limited settings such as Bangladesh.

**Methods:**

During 2019, we conducted a population-based observational study in Dhaka (surveillance site of icddr,b). We collected stool samples from randomly selected adults and tested for Enterobacterales with extended-spectrum cephalosporin resistance (ESCrE) and carbapenem resistance (CRE) using selective media followed by VITEK-2 confirmation. Participants completed demographic surveys assessing food consumption, animal contact, sanitation, water sources, and healthcare exposure. We identified factors associated with ESCrE and CRE colonization using bivariable and multivariable logistic regression, adjusting for potential confounders and clustering.

**Results:**

Of 714 enrolled individuals, 557 (78%) were colonized with ESCrE and 66 (9%) with CRE. In bivariable analysis, factors associated with ESCrE colonization included fresh fruit consumption in the past week (OR 1.8, 95% CI 1.2-2.9), public tap as main source of drinking water compared with basic improved source (OR 3.3, 1.0-11.0), and unimproved toilet (pour/flush to open drain, pit latrine without slab) compared with basic improved toilet (OR 11, 2.9-42.0). Only consumption of fresh fruit was significant in the multivariable analysis (aOR 2.0, 1.3-3.2). Factors associated with CRE colonization in bivariable analysis were hospitalization in the last 3 months (OR 3.2, 1.2-8.6), limited sanitation facility (improved toilet shared with other households) (OR 2.0, 1.0-3.8), and limited hygiene (availability of handwashing facility on premises without soap and/or water) (OR 3.3, 1.4-7.7). The only factor that was significant in multivariable analysis was hospitalization in the last 3 months (aOR 3.0, 1.0-8.7).

**Conclusion:**

While ESCrE colonization is common in urban communities, hospitals may be contributing to community spread of CRE. Targeted interventions focused on healthcare facilities may be needed to mitigate the transmission of AMR organisms.

**Disclosures:**

**All Authors**: No reported disclosures.

